# Supplementary material for: Convergent Within-Host Adaptation of Pseudomonas aeruginosa through the Transcriptional Regulatory Network
Source: mSystems. 2023 Mar 28;8(2):e00024-23. doi: 10.1128/msystems.00024-23 (PMC10134825; doi:10.1128/msystems.00024-23)
Supplement: TEXT S1 [file msystems.00024-23-s0001.docx]

**Supplemental Information**

**Convergent within-host adaptation of *Pseudomonas aeruginosa* through the transcriptional regulatory network**

**Yair E. Gatt, Dana Savion, Tal Bamberger and Hanah Margalit**

Department of Microbiology and Molecular Genetics, Institute for Medical Research Israel-Canada, Faculty of Medicine, The Hebrew University of Jerusalem, Jerusalem 9112102, Israel

**Supplementary Results**

**Determining the phylogenetic relationship between the isolates using TRACE**

The progenitor-progeny pairs used in this study were generated by the TRACE algorithm, an algorithm developed and used in Gatt and Margalit (1) to determine the phylogenetic relationships between the isolates of each strain. Briefly, all the isolates derived from a single patient were defined as same-strain isolates. In the first step of the phylogenetic analysis, all isolates of a single strain are classified to clones, resulting in a classification that is nearly identical to their classification by Multilocus Sequence Typing (MLST) profiles. Most strains only include a single clone, i.e. a monoclonal infection. In the following step, we assess different phylogenetic trees where both the leaves of the tree and the internal nodes represent the different isolates, and branches can only connect isolates to isolates from later time points. The best possible tree for each clone is determined based on having the shortest branches, which are the genetic distances between the isolates as determined by either *breseq* or kSNP (2-5). Finally, we assign each branch in the best tree a confidence score, by computing the fraction of trees in which the branch appears among the 10,000 best possible trees for the clone. Each branch with a confidence score >0.4 is considered as corresponding to a high confidence progenitor-progeny pair, which are recorded for further analyses.

**Convergent adaptation at the transcriptional level is predicted to lead to reduced expression of genes related to HCN synthesis**

We searched for genes with high Heterogeneity Score (HS) and extreme mROS, indicating convergent adaptation of these genes at the transcriptional level. Genes with HS>2.5 were determined as having high HS (based on the distribution of the HS values across all genes, see Fig. 3b in the main text). Focusing on 118 genes with both high HS and extreme mROS (|mROS| > 0.15), the expression of 26 genes, including 24 non-hypothetical genes, is predicted to consistently increase, and the expression of 92 genes, including 61 non-hypothetical genes, is predicted to consistently decrease. For each of these two groups we studied enrichment by specific KEGG pathways or GO annotations using hypergeometric tests, while correcting for the testing of multiple hypotheses using the Benjamini-Hochberg method (6).

The group whose expression is predicted to increase is statistically significantly enriched with genes related to β-lactam resistance, which is in-line with the increased antibiotic resistance of *Pseudomonas aeruginosa* during chronic infection. It is also enriched with GO annotations and KEGG pathways linked to glycine and the closely related sarcosine, including glycine, serine and threonine metabolism, dimethylglycine dehydrogenase and oxidase activity, glycine betaine catabolism to which we refer in the main manuscript, sarcosine oxidase activity, and sarcosine catabolic process. It is also enriched with genes corresponding to two additional GO annotations – tetrahydrofolate metabolic process and oxidation-reduction process.

The group whose expression is predicted to decrease is enriched with genes related to the KEGG pathways Histidine metabolism and Cyanoamino acid metabolism. The latter pathway includes genes related to the synthesis of hydrogen cyanide. This indicates selection for reduced synthesis of HCN during infections, achieved in different strains by different LOF events in genes encoding transcriptional regulators. Indeed, HCN is known not to be produced under anaerobic conditions (7, 8), which are prevalent in the biofilms common in chronic *P. aeruginosa* infection (9). Genes in this pathway are positively regulated by the master quorum-sensing regulator LasR (10), which frequently undergoes LOF during infection (Fig. S1b). While the reduced synthesis of HCN can be achieved by LOF mutations in *lasR*, the high HS of these genes demonstrates that the same result could be achieved by LOF mutations in other TF/SF-encoding genes. This group is also enriched with GO annotations related to pyoverdine and PQQ synthesis, which is in line with our previous findings, and to glycine dehydrogenase activity.

**Predicted changes in expression explain changes in antibiotic resistance that were not explained by genetic changes**

To verify our predictions regarding antibiotic resistance, we searched for strains that exhibited changes in Minimum Inhibitory Concentrations (MIC) that could not be explained by any genetic changes identified in the original studies. We found 13 strains where antibiotic resistance was systematically examined in the original study for different antibiotic classes. Out of these 13 strains, nine passed the quality thresholds of Gatt and Margalit (1) and had predictions of genes undergoing LOF during infection. In four of the nine strains, at least one transcriptional regulator underwent LOF during infection.

All four of these strains had changes in MIC for which the original authors could not find an explanation using the accumulated mutations between the isolates. We suggest possible explanations for the changes in the MIC for three of the strains through the transcriptional regulatory network (TRN). The study and patient numbers refer to their numbering in Gatt and Margalit (1) and can also be found in Table S1.

Study 108 patient 4 (11)

This patient suffered from VAP (Ventilator-associated pneumonia) caused by *P. aeruginosa*. A day 2 isolate of the infecting strain was found to be the progenitor of two closely associated isolates at days 21 and 25. The progeny isolates have increased sensitivity to some β-lactams (ceftazidime and imipenem) and greatly increased sensitivity to tobramycin. No mutation in relevant genes in the isolates was found to explain this phenotype.

Through the network, we predicted reduced expression of *ndvB*, a gene that, when undergoing LOF, was shown to be associated with increased sensitivity to tobramycin in biofilms (12, 13). Additionally we predicted reduced expression of the *PA1874-PA1877* operon, encoding an ABC transporter whose deletion was also found to lead to sensitivity to tobramycin (14). Another finding that can explain the phenotype is the increased expression of *nalC*. NalC is a repressor of the MexAB-OprM efflux pump in *P. aeruginosa*, which is key to resistance to β-lactams (15-19). A fourth prediction in-line with the phenotype is the reduced expression of *mexD*, encoding part of the MexCD-OprJ efflux pump, which is related to resistance to β-lactams via efflux (15-19).

Study 46, patient 2 (20)

Gatt and Margalit (1) defined three progenitor-progeny pairs in this Cystic Fibrosis (CF) patient suffering from chronic *P. aeruginosa* infection. The first time point isolate was the progenitor for the isolates of time points 2, 3 and 4. For the progenitor-progeny pair between the first and third time points, the strain became more resistant to several classes of antibiotics including β-lactams. The greatest change was seen for aztreonam, which genetic mutations could not explain. López-Causapé et al. (20) hypothesized that resistance may be related to increased expression of the MexAB-OprM efflux pump as it secretes β-lactamases and has high affinity for aztreonam. Indeed, in our data we predicted increased expression of this pump, explaining the resistance phenotype.

The strain developed increased resistance to nearly all examined antibiotics between the first and fourth time point isolates. Despite this, no mutation related to antibiotic resistance was observed. We predicted decreased expression of *PA1942*. Madden et al. (21) recently implicated the LOF of this gene in resistance to meropenem and piperacillin, explaining some of the phenotype. Additionally, we predicted reduced expression of *PA2491*. LOF of this gene has been linked to resistance to chloramphenicol, quinolones and imipenem, explaining some of the phenotype as well (22). It should be noted that this resistance was found to be mediated via both the MexEF-OprJ efflux pump and decreased OprD production, and in our data the *mexEF* genes were predicted to have reduced expression.

Study 46 patient 3 (20)

Gatt and Margalit (1) defined two progenitor-progeny pairs in this CF patient suffering from chronic *P. aeruginosa* infection. The first time point isolate was the progenitor for the isolates of time points 3 and 4. For the progenitor-progeny pair between the first and third time points, the strain became more sensitive to nearly all tested antibiotics. The strain gained several mutations associated with antibiotic resistance, but none that could explain the increased sensitivity. In our data we predicted increased expression of *nalD*, a repressor of the MexAB-OprM efflux pump, which may explain some of the phenotype. This interaction is not found in the TRN we used (23), which explains why the *mexAB-oprM* genes themselves were not predicted to have a negative ROS.

**Clustering analysis reveals independent gene pairs within the same clusters**

When clustering the different genes based on their ROS vectors in the different progenitor-progeny pairs, we found that many genes found in the same cluster are simply co-regulated by the same transcriptional regulator. To filter out these genes, we searched for clusters including independent pairs of genes – genes that do not have a shared transcriptional regulator (ancestor) in a distance less than four edges from the two genes in the network. We also required that the independent pairs have |mROS|>0.15. Two clusters included multiple such independent pairs – Cluster 13 and Cluster 14. Cluster 14 is described in the main text. Cluster 13 includes dozens of independent pairs, suggesting it represents an adaptive strategy with transcriptome-wide changes, similarly to Cluster 14. While Clusters 13 and 14 present similar scores for some pathways, many known pathways associated with host-adaptation of *P. aeruginosa* change in accordance with their known role in adaptation in Cluster 14 but are changed in the opposite direction or not at all in Cluster 13. This includes pathways related to alginate production, anaerobic respiration, fatty acid metabolism, degradation of superoxide radicals, O-antigen biosynthesis and sulfate assimilation. Similarly, siderophore and heme biosynthesis are decreased in Cluster 14, with no change in heme biosynthesis in Cluster 13 and a predicted decrease in pyochelin and increase in pyoverdine biosynthesis. *P. aeruginosa* strains are known to become auxotrophic for amino acids during adaptation (24). According to the analysis of PseudoCyc pathways (Table S4), both clusters are predicted to have increased synthesis or degradation of various amino acids, but also a predicted increased synthesis or decreased degradation of others.

*P. aeruginosa* is known to undergo an intriguing change in central metabolism during host adaptation. The expression of the *P. aeruginosa* pyruvate dehydrogenase is often decreased within the CF airways, leading to a split between the glycolysis pathway and the TCA cycle. The TCA cycle then continues to operate via the glyoxylate shunt, which utilizes amino acids and lactate (25). This is in accordance with Cluster 13, which includes a predicted decrease in pyruvate fermentation and a predicted increase in the TCA cycle, lactate oxidation, glycolysis and the glyoxylate cycle. On the other hand, Cluster 14 is associated with an increase in pyruvate oxidation and a decrease in the TCA cycle.

**References**

1. Gatt YE, Margalit H. 2021. Common Adaptive Strategies Underlie Within-Host Evolution of Bacterial Pathogens. Molecular Biology and Evolution 38:1101-1121.

2. Barrick JE, Colburn G, Deatherage DE, Traverse CC, Strand MD, Borges JJ, Knoester DB, Reba A, Meyer AG. 2014. Identifying structural variation in haploid microbial genomes from short-read resequencing data using *breseq*. BMC Genomics 15:1039.

3. Deatherage DE, Barrick JE. 2014. Identification of mutations in laboratory-evolved microbes from next-generation sequencing data using *breseq*. Methods in Molecular Biology 1151:165-188.

4. Gardner SN, Hall BG. 2013. When whole-genome alignments just won't work: kSNP v2 software for alignment-free SNP discovery and phylogenetics of hundreds of microbial genomes. PLoS ONE 8:e81760.

5. Gardner SN, Slezak T, Hall BG. 2015. kSNP3. 0: SNP detection and phylogenetic analysis of genomes without genome alignment or reference genome. Bioinformatics 31:2877-2878.

6. Benjamini Y, Hochberg Y. 1995. Controlling the False Discovery Rate: A Practical and Powerful Approach to Multiple Testing. Journal of the Royal Statistical Society Series B (Methodological) 57:289-300.

7. Castric P. 1994. Influence of oxygen on the *Pseudomonas aeruginosa* hydrogen cyanide synthase. Current Microbiology 29:19-21.

8. Castric PA. 1975. Hydrogen cyanide, a secondary metabolite of *Pseudomonas aeruginosa*. Canadian Journal of Microbiology 21:613-618.

9. Yoon SS, Hennigan RF, Hilliard GM, Ochsner UA, Parvatiyar K, Kamani MC, Allen HL, DeKievit TR, Gardner PR, Schwab U, Rowe JJ, Iglewski BH, McDermott TR, Mason RP, Wozniak DJ, Hancock RE, Parsek MR, Noah TL, Boucher RC, Hassett DJ. 2002. *Pseudomonas aeruginosa* anaerobic respiration in biofilms: relationships to cystic fibrosis pathogenesis. Developmental Cell 3:593-603.

10. Pessi G, Haas D. 2000. Transcriptional Control of the Hydrogen Cyanide Biosynthetic Genes *hcnABC* by the Anaerobic Regulator ANR and the Quorum-Sensing Regulators LasR and RhlR in *Pseudomonas aeruginosa*. Journal of Bacteriology 182:6940-6949.

11. Wang K, Chen Y-Q, Salido MM, Kohli GS, Kong J-L, Liang H-J, Yao Z-T, Xie Y-T, Wu H-Y, Cai S-Q, Drautz-Moses DI, Darling AE, Schuster SC, Yang L, Ding Y. 2017. The rapid *in vivo* evolution of *Pseudomonas aeruginosa* in ventilator-associated pneumonia patients leads to attenuated virulence. Open Biology 7:170029.

12. Beaudoin T, Zhang L, Hinz AJ, Parr CJ, Mah TF. 2012. The biofilm-specific antibiotic resistance gene *ndvB* is important for expression of ethanol oxidation genes in *Pseudomonas aeruginosa* biofilms. Journal of Bacteriology 194:3128-3136.

13. Mah TF, Pitts B, Pellock B, Walker GC, Stewart PS, O'Toole GA. 2003. A genetic basis for *Pseudomonas aeruginosa* biofilm antibiotic resistance. Nature 426:306-310.

14. Zhang L, Mah TF. 2008. Involvement of a novel efflux system in biofilm-specific resistance to antibiotics. Journal of Bacteriology 190:4447-4452.

15. Drissi M, Ahmed ZB, Dehecq B, Bakour R, Plésiat P, Hocquet D. 2008. Antibiotic susceptibility and mechanisms of β-lactam resistance among clinical strains of *Pseudomonas aeruginosa*: First report in Algeria. Médecine et Maladies Infectieuses 38:187-191.

16. Okamoto K, Gotoh N, Nishino T. 2002. Alterations of susceptibility of *Pseudomonas aeruginosa* by overproduction of multidrug efflux systems, MexAB-OprM, MexCD-OprJ, and MexXY/OprM to carbapenems: Substrate specificities of the efflux systems. Journal of Infection and Chemotherapy 8:371-373.

17. Pai H, Kim J, Kim J, Lee JH, Choe KW, Gotoh N. 2001. Carbapenem resistance mechanisms in *Pseudomonas aeruginosa* clinical isolates. Antimicrob Agents Chemother 45:480-484.

18. Poole K. 2011. *Pseudomonas aeruginosa*: resistance to the max. Frontiers in Microbiology 2:65.

19. Tomás M, Doumith M, Warner M, Turton JF, Beceiro A, Bou G, Livermore DM, Woodford N. 2010. Efflux Pumps, OprD Porin, AmpC β-Lactamase, and Multiresistance in *Pseudomonas aeruginosa* Isolates from Cystic Fibrosis Patients. Antimicrobial Agents and Chemotherapy 54:2219-2224.

20. López-Causapé C, Sommer LM, Cabot G, Rubio R, Ocampo-Sosa AA, Johansen HK, Figuerola J, Cantón R, Kidd TJ, Molin S, Oliver A. 2017. Evolution of the *Pseudomonas aeruginosa* mutational resistome in an international Cystic Fibrosis clone. Scientific Reports 7:5555.

21. Madden DE, Baird T, Bell SC, McCarthy KL, Price EP, Sarovich DS. 2022. Keeping up with the pathogens: Improved antimicrobial resistance detection and prediction in *Pseudomonas aeruginosa*. medRxiv doi:10.1101/2022.08.11.22278689:2022.08.11.22278689.

22. Sobel ML, Neshat S, Poole K. 2005. Mutations in *PA2491 (mexS)* promote MexT-dependent *mexEF-oprN* expression and multidrug resistance in a clinical strain of *Pseudomonas aeruginosa*. Journal of Bacteriology 187:1246-1253.

23. Galán-Vásquez E, Luna-Olivera BC, Ramírez-Ibáñez M, Martínez-Antonio A. 2020. RegulomePA: a database of transcriptional regulatory interactions in *Pseudomonas aeruginosa* PAO1. Database 2020.

24. Behrends V, Ryall B, Zlosnik JE, Speert DP, Bundy JG, Williams HD. 2013. Metabolic adaptations of *Pseudomonas aeruginosa* during cystic fibrosis chronic lung infections. Environmental Microbiology 15:398-408.

25. Rossi E, La Rosa R, Bartell JA, Marvig RL, Haagensen JAJ, Sommer LM, Molin S, Johansen HK. 2021. *Pseudomonas aeruginosa* adaptation and evolution in patients with cystic fibrosis. Nature Reviews Microbiology 19:331-342.
