# Supplementary figures and images for: Convergent Within-Host Adaptation of Pseudomonas aeruginosa through the Transcriptional Regulatory Network
Source: mSystems. 2023 Mar 28;8(2):e00024-23. doi: 10.1128/msystems.00024-23 (PMC10134825; doi:10.1128/msystems.00024-23)

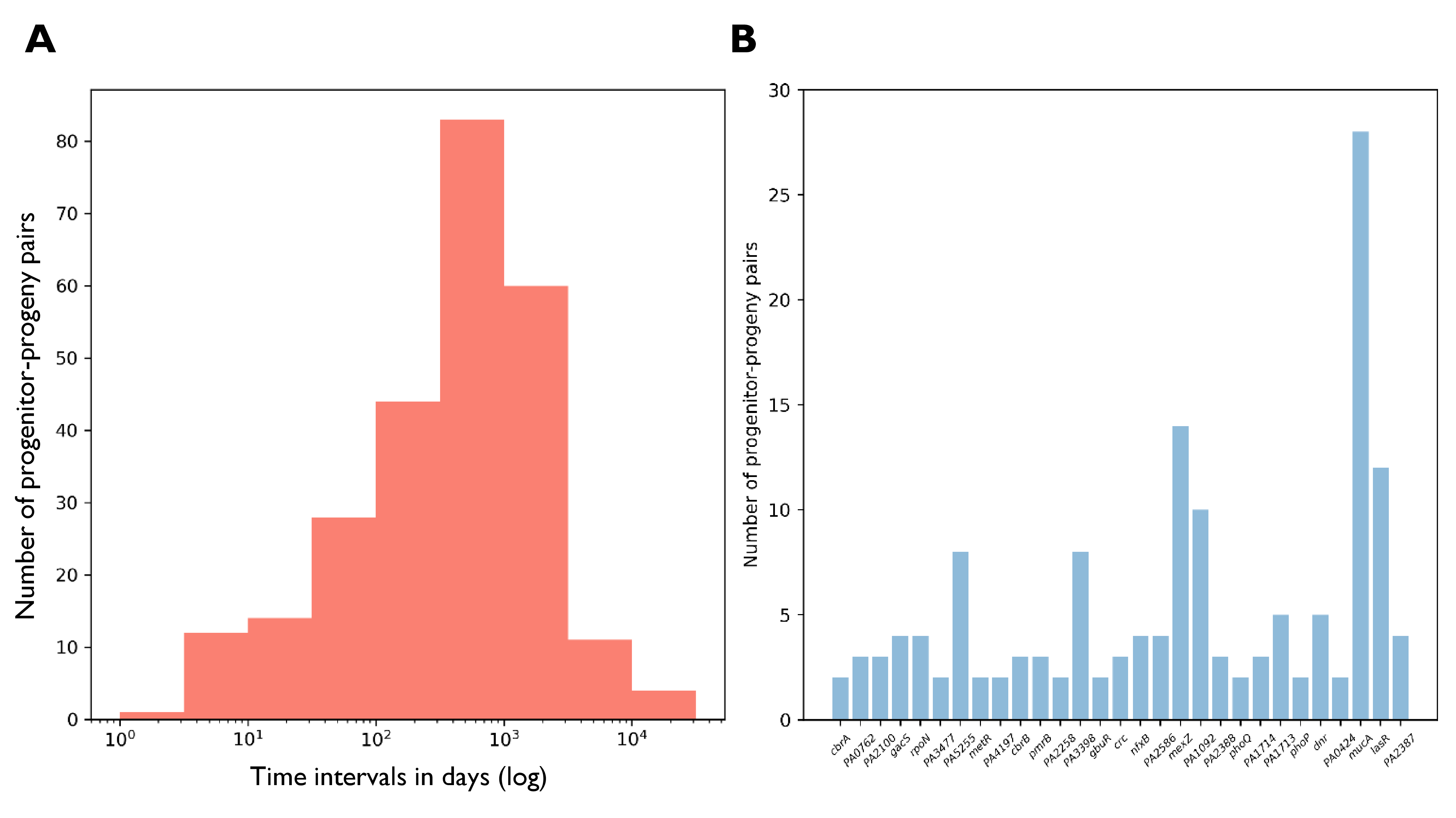

Supplement: FIG S1 [file msystems.00024-23-s0006.tif]

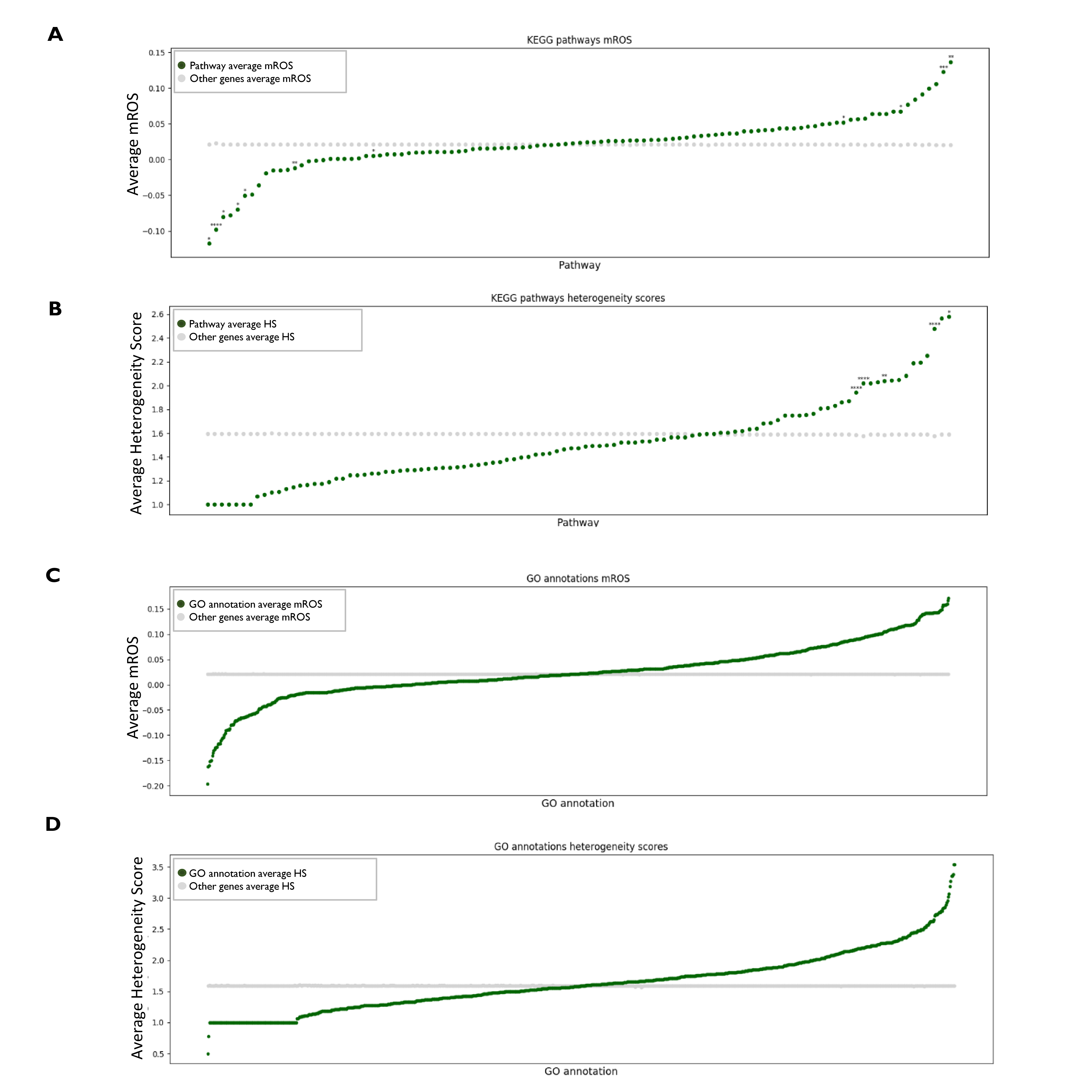

Supplement: FIG S2 [file msystems.00024-23-s0007.tif]

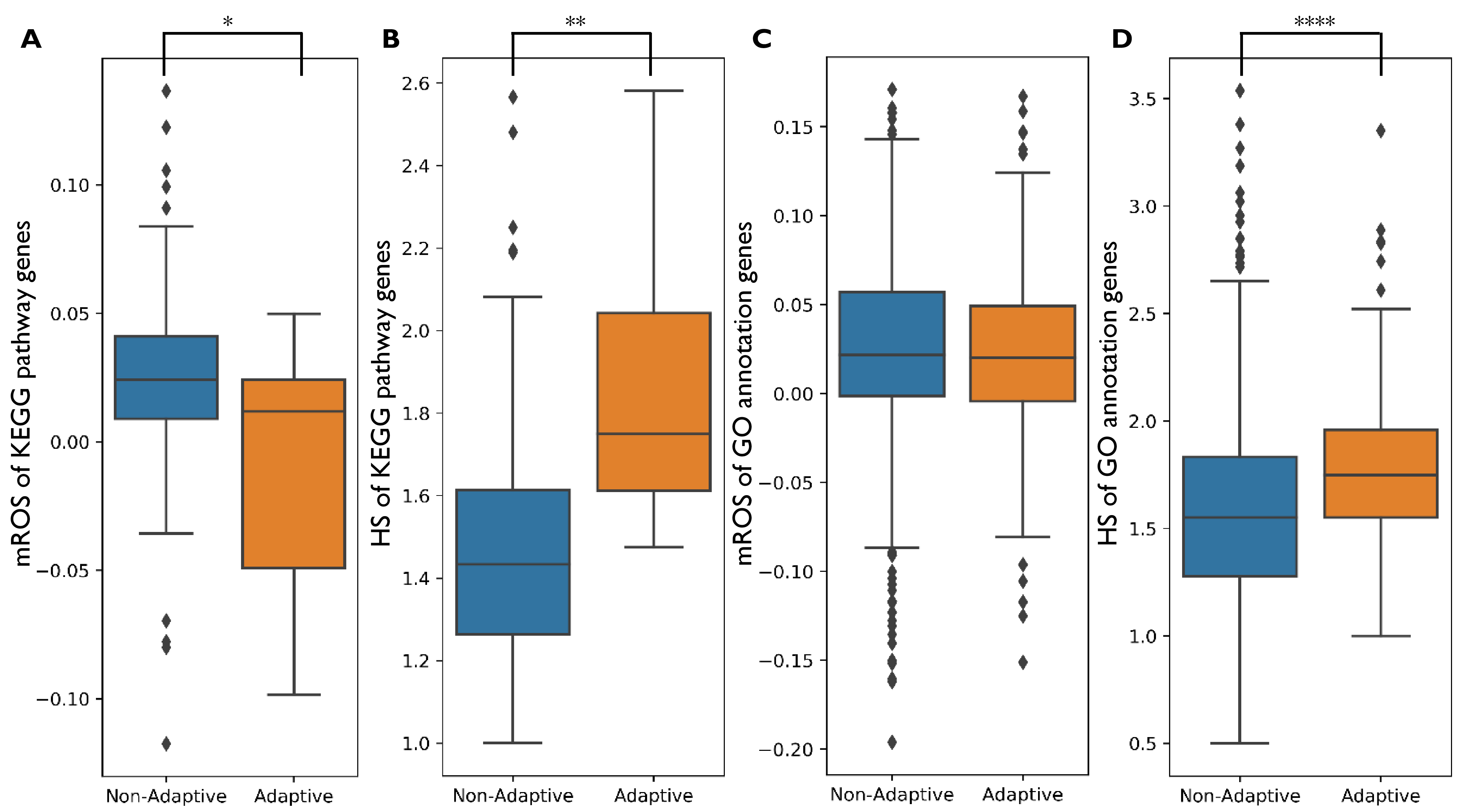

Supplement: FIG S3 [file msystems.00024-23-s0008.tif]

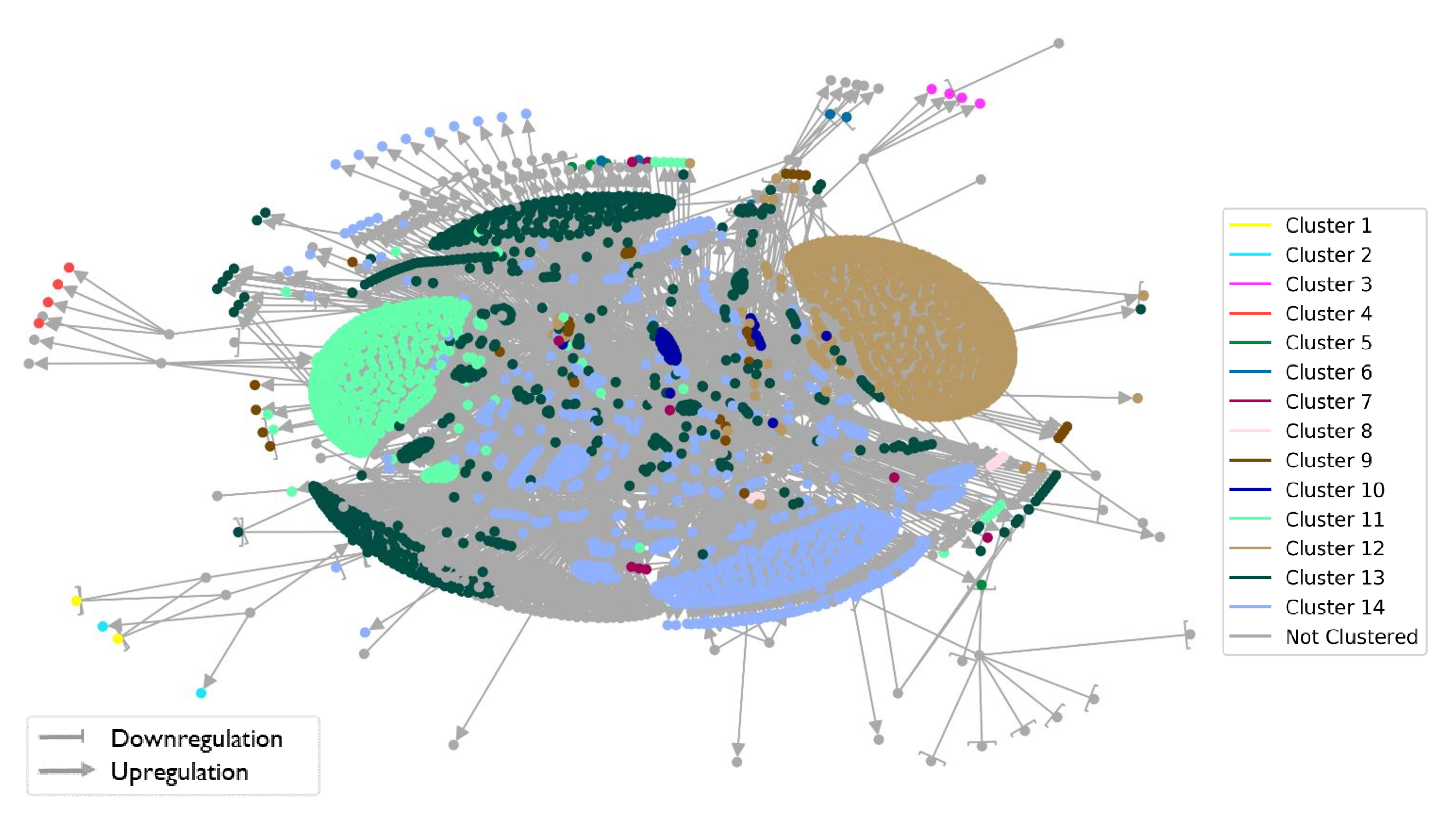

Supplement: FIG S4 [file msystems.00024-23-s0009.tif]
